# DCM for complex-valued data: Cross-spectra, coherence and phase-delays

**DOI:** 10.1016/j.neuroimage.2011.07.048

**Published:** 2012-01-02

**Authors:** K.J. Friston, A. Bastos, V. Litvak, K.E. Stephan, P. Fries, R.J. Moran

**Affiliations:** a The Wellcome Trust Centre for Neuroimaging, University College London, Queen Square, London WC1N 3BG, UK; b Center for Neuroscience and Center for Mind and Brain, University of California-Davis, Davis, CA 95618, USA; c Ernst Strüngmann Institute in Cooperation with Max Planck Society, Deutschordenstraße 46, 60528 Frankfurt, Germany; d Laboratory for Social and Neural Systems Research, Dept. of Economics, University of Zurich, Switzerland

## Abstract

This note describes an extension of Bayesian model inversion procedures for the Dynamic Causal Modeling (DCM) of complex-valued data. Modeling complex data can be particularly useful in the analysis of multivariate ergodic (stationary) time-series. We illustrate this with a generalization of DCM for steady-state responses that models both the real and imaginary parts of sample cross-spectra. DCM allows one to infer underlying biophysical parameters generating data (like synaptic time constants, connection strengths and conduction delays). Because transfer functions and complex cross-spectra can be generated from these parameters, one can also describe the implicit system architecture in terms of conventional (linear systems) measures; like coherence, phase-delay or cross-correlation functions. Crucially, these measures can be derived in both sensor and source-space. In other words, one can examine the cross-correlation or phase-delay functions between hidden neuronal sources using non-invasive data and relate these functions to synaptic parameters and neuronal conduction delays. We illustrate these points using local field potential recordings from the subthalamic nucleus and globus pallidus, with a special focus on the relationship between conduction delays and the ensuing phase relationships and cross-correlation time lags between population activities.

## Highlights

► We use DCM to predict coherence and phase-differences in time-series recordings. ► We generalize variational Bayesian techniques for application to complex data. ► Conventional time-series measures are imbued with plausible biophysical form. ► We highlight differences in source and sensor level descriptors.

## Introduction

This technical note describes a dynamic causal or generative model for time-series, under ergodic assumptions. It is based on a linearization of mean-field models of coupled dynamical systems; in our case, neuronal subpopulations. Under the assumption that the system is driven by exogenous fluctuations with known (or parametric) spectral densities and uniform phase-distributions, it is possible to predict the coherence and phase-differences observed among system responses; in our case electrophysiological measurements. This enables the model to be optimized using empirical measures of (complex) cross-spectral densities and thereby access hidden parameters governing the data-generating process (e.g., coupling parameters and rate constants). We validate this model using simulations and illustrate its application using real local field potential (LFP) data.

The contributions of this work are threefold. First, it generalizes variational Bayesian techniques (Variational Laplace: Friston et al., 2007) used to invert models of empirical data so that they can be applied to complex data. This generalization can be applied in any setting that uses variational Laplacian schemes to select and optimize models. It is illustrated here in the context of Dynamic Causal Modeling of steady-state responses in electrophysiology. Second, we use the generalization to show how conventional time-series measures of activity and coupling in the frequency domain can be derived from Dynamic Causal Models that have a biophysically plausible form. Finally, we show how this enables the computation of conventional measures, such as coherence and phase-delay functions, between hidden states; in other words, between sources as opposed to sensors.

Current applications of Dynamic Causal Modeling have been used to explain real-valued data features, including evoked transients, induced responses and power spectra (Kiebel et al., 2008a,b), using biologically informed mean-field models of coupled dynamical systems. The ability to model complex-valued data features offers two key advantages. First, while current DCMs can estimate conduction delays, the estimates do not have access to phase information. In principle, models that can predict or generate complex data enable the phase relationships among observed responses to inform and constrain estimates of model parameters, like conduction delays. More importantly, the extension to complex valued data bridges the technical divide between model-based and model-free analyses (Brown et al., 2004; Kay and Marple, 1981), as we hope to show.

The generalization to complex data was motivated by DCM for steady-state responses, as applied to electrophysiological time-series acquired under particular brain states (Moran et al., 2007, 2008). Our focus here is on the potential importance of complex-valued data features and the implications for the inversion or optimization of models of those features. We try to illustrate the potential of this scheme by looking at how precisely axonal conduction delays can be estimated, when fitting complex cross-spectra, in relation to real-valued cross-spectra. More generally, we anticipate that this DCM will provide a useful link between the generative modeling of biological time-series and conventional (linear systems) characterizations (e.g. Kay and Marple, 1981) that predominate in electrophysiology. This link rests upon the fact that conventional measures (e.g., coherence, phase-delay and auto-correlation functions) are caused by neuronal circuits with particular biophysical parameters (e.g., synaptic efficacy, time constants and conduction delays). This means that biophysical parameter estimates can be used to create conditional estimates of coherence and cross-correlation functions. In turn, this means that it is possible to infer which biophysical parameters are responsible for observed coherence or phase-delays. Conceptually, the difference between DCM and conventional measures of coupling (i.e., functional connectivity) lies in the fact that DCM appeals to an explicit generative or forward model of how data features are caused (i.e., effective connectivity). In this instance, the data features are provided by the spectral behavior of observed time-series that are, usually, the end point of conventional analyses. The advantage of having an underlying generative model is that one can estimate the spectral behavior and relationships, not just among observed sensors or data channels, but between the neuronal sources generating those data. Furthermore, one can map quantitatively between the underlying biophysical parameters and spectral summaries. We will illustrate these points using simulated and real data.

Dynamic Causal Modeling for steady-state responses has been used to make inferences about hidden neuronal states and parameters using both invasive and non-invasive data. There has been a considerable effort to validate this approach using simulations, developmental manipulations and psychopharmacological interventions (Moran et al., 2008, 2009). There is now a large literature on Dynamic Causal Modeling in electrophysiology (Chen et al., 2008; Daunizeau et al., 2009; David et al., 2006a,b; Kiebel et al., 2007) and, in particular, models for steady-state activity (Moran et al., 2007, 2008, 2009, 2011). We take DCM for steady-state responses as the starting point for the causal modeling of complex-valued data. This is because this current scheme uses the absolute value of the cross-spectrum between channels. However, the cross-spectrum is a complex quantity, which means that it has the attributes of modulus (absolute value or amplitude) and argument (angle or phase). This means that one is throwing away information when using absolute measures to invert or fit generative models. In what follows, we look at the advantages of using both amplitude and phase information.

The phase of the cross-spectrum is usually taken to indicate something about systematic lags or delays between two signals. If one signal appears in the other, after a constant time lag, then the phase-difference scales with frequency. In practice, this notion has been used, for example, in epilepsy research where authors have used phase differences between signals from different intracerebral electrodes or EEG channels to estimate conduction or propagation delays (Brazier, 1972; Gotman, 1981). A regression of the phase-difference on frequency is also often used to estimate temporal delays over a particular frequency range (Rosenberg et al., 1989). More generally, one can divide the phase-difference by frequency to quantify time lags as a function of frequency. A further advantage of working with the complex cross-spectrum is that its inverse Fourier transform provides the cross-correlation function between two time-series. If the correlation structure is dominated by a single time delay, the latency of this delay can often be inferred from the timing of a peak in the cross correlation. In short, a generative model of complex-valued data features (e.g. cross-spectra) provides a more complete model of data and provides conditional estimates of coherence, phase-delay and cross-correlation functions that are implicitly constrained by the functional architectures inducing those correlations. The examples in this paper attempt to illustrate this in a practical fashion.

This note comprises four sections. In the first, we consider the nature of cross-spectra and their relationship to coherence and phase-delays. This section is used to frame conventional measures of coupling in terms of the underlying transfer functions between sources generating data. Crucially it shows that although coherence is sensitive to the dispersion of phase-differences between two sources or sensors, coherence does not provide a complete picture of coupling, because it is insensitive to phase-delays *per se*. As indicated above, a more comprehensive summary rests upon the complex cross-spectra that include both real and imaginary parts that embody phase-delays. However, the phase-delay does not report the time-delays between two signals directly and can only be interpreted in relation to a model of how time-delays are manifest in data. The models we use here are biologically plausible (neural mass) models, based on delay differential equations that make time-delays an explicit model parameter. This section concludes with a brief description of these neural mass models that constitute a generative model for steady-state responses. The second section briefly reviews the inversion of these models, with a special focus on constructing free-energy bounds on model log-evidence for complex-valued data. In the third section, we present an illustrative analysis of simulated data, in which we know the coupling strengths and time-delays among a small number of neuronal populations. These simulations are used to verify that the various conditional estimates of coupling, in time and frequency space, can recover the true values in a reasonably precise way. Our special interest here is in the direction of coupling and conduction delays, as inferred through the conditional distribution over time-delays and how they manifest in phase-delay and cross-correlation functions. There are many interesting aspects of the mapping between model parameters (effective connectivity) and spectral characterizations (functional connectivity): we have chosen conduction delays as one of the more prescient. This is because there is a non-trivial relationship between (axonal) conduction delays and delays that manifest in terms of phase-delays and cross-correlation lags. In the final section, we repeat the analysis of the third section but using real data from local field potential recordings in rats.

## Coherence and causal modeling

In this section, we look at the nature of conventional measures of functional connectivity from the point of view of phase-differences and their distribution. The main points made in this treatment are: (i) Coherence is a function of the absolute value of the cross-spectra and, as such, provides an incomplete picture of spectral dependencies among stationary time-series. (ii) Furthermore, even if we consider complex cross-spectra, their phase information cannot be interpreted in terms of time-delays, unless we know (or model) how they were caused. Specifically, when the coupling between two sources of data is bidirectional, there is no straightforward correspondence between phase and time-delays. The resulting ambiguity can only be resolved by reference to a model of how phase-differences are generated. This section concludes by briefly reprising the generative models used in DCM of steady-state responses that resolve this ambiguity.

### Theoretical background

#### Notation and preliminaries

Where possible, we will denote Fourier transforms by upper case letters such that *S*_*i*_(*ω*) ∈ *C* is the Fourier transform of a stationary time-series $s_{i}(t)\in R$. We will also make a crucial distinction between the observed or sample cross-spectrum *g*_*ij*_(*ω*) (referred to as the sample cross-spectrum) and a cross-spectrum *g*_*ij*_(*ω*, *θ*) predicted by a model with parameters *θ* (referred to as the modeled cross-spectrum). Unless used as a subscript, $j=\sqrt{-1}$.

#### Coherence and phase-synchronization

The complex coherence function between two wide-sense stationary signals *s*_*i*_(*t*) and *s*_*j*_(*t*), is equal to their cross power spectrum *g*_*ij*_(*ω*) = 〈*S*_*i*_*S*_j_*〉 ∈ *C* divided by the square root product of the two auto power spectra (Carter, 1987; Priestley, 1981). The magnitude-squared coherence (Carter, 1987), $C_{\mathrm{ij}}(\omega )\in R$ (herein referred to as simply ‘coherence’) is given by:

1 $$C_{\mathrm{ij}}=\frac{{\left|g_{\mathrm{ij}}(\omega )\right|}^{2}}{g_{\mathrm{ii}}(\omega )g_{\mathrm{jj}}(\omega )}\in R$$

The coherence can be factorized into the correlation between the signal amplitudes and the (circular) dispersion of their phase-differences (e.g. Priestley, 1981; Friston et al., 1997, (Eq. A3)):

2 $$\begin{array}{c} C_{\mathrm{ij}}=\frac{{\langle \alpha_{i}\alpha_{j}\rangle }^{2}}{\langle \alpha_{i}^{2}\rangle \langle \alpha_{j}^{2}\rangle }\times \Phi_{\mathrm{ij}}\\ \Phi_{\mathrm{ij}}={\left(\int_{-\pi }^{\pi }p(\delta_{\mathrm{ij}})\sin(\delta_{\mathrm{ij}})d\delta_{\mathrm{ij}}\right)}^{2}+{\left(\int_{-\pi }^{\pi }p(\delta_{\mathrm{ij}})\cos(\delta_{\mathrm{ij}})d\delta_{\mathrm{ij}}\right)}^{2} \end{array}$$

Here, *α*_*i*_ = |*S*_*i*_| corresponds to signal amplitude and *ϕ*_*i*_ = arg{*S*_*i*_} to phase (where radial frequency is $\omega :={\dot{\phi }}_{i}$). Eq. (2) shows that coherence depends on a function of the density *p*(*δ*_*ij*_) over phase-differences: *δ*_*ij*_ = *φ* _*i*_ − *φ* _*j*_. This function Φ(*ω*)_*ij*_ is called the phase-synchronization index (Mardia and Jupp, 1999; Pascual-Marqui, 2007) and reflects the circular variance or dispersion of phase-differences. Eq. (2) means that coherence is effectively the normalized absolute value of the cross-spectrum, while phase-synchronization is the absolute value of the cross-spectrum when derived from normalized Fourier transforms (see Pascual-Marqui, 2007 for a generalization to multivariate time-series). The key thing to note here is that coherence does not change with the average phase-difference, only its dispersion; that is, coherence reflects the stability of the phase difference.

In what follows, we will be concerned with generative models of data features that disclose the mapping between some exogenous (neuronal) fluctuations or innovations $u_{k}(t)\in R$ with Fourier Transform *U*_*k*_(*ω*) ∈ *C* and observable signals $s_{i}(t)\in R$, under ergodic assumptions. Under linear assumptions, this mapping is specified by a kernel, *κ*_*i*_^*k*^(*τ*, *θ*) = ∂ *s*_*i*_(*t*)/∂ *u*_*k*_(*t* − *τ*), whose parameters *θ* we wish to estimate. Usually, one would associate each innovation with a neuronal population or source of signals (although there may be others, like common input). The kernels are then defined by a model of how the innovations are transformed by synaptic processing in connected sources and the physical transmission of source activity to one or more sensors. Hence, the parameters of the model (or the kernels) include the effective connectivity among sources (with time-delays) and the parameters of any mapping from sources to channels (e.g., an electromagnetic forward model for EEG data).

The cross-spectral density is the sum of cross-spectra induced by each innovation, where there is an innovation for each source of activity that contributes to observed signals. The cross spectrum due to the *k*-th innovation is simply the product of the transfer functions (Fourier transforms) of the corresponding kernels, *K*_*i*_^*k*^(*ω*, *θ*) and the spectral density, *γ*_*k*_(*ω*) = 〈|*U*_*k*_||*U*_*k*_|〉 of (statistically independent) innovations

3 $$\begin{array}{c} g_{\mathrm{ij}}(\omega ,\theta )=\langle \sum_{\mathrm{kl}}|K_{i}^{k}||K_{j}^{l}|\cdot \text{exp}(j(\phi_{i}^{k}-\phi_{j}^{l}))\cdot |U_{k}||U_{l}|\cdot \text{exp}(j(\phi^{k}-\phi^{l}))\rangle \\ =\sum_{\mathrm{kl}}|K_{i}^{k}||K_{j}^{l}|\cdot \text{exp}(j(\phi_{i}^{k}-\phi_{j}^{l}))\cdot \langle \left|U_{k}||U_{l}|\cdot \text{exp}(j(\phi^{k}-\phi^{l})\right)\rangle \\ =\sum_{k}|K_{i}^{k}||K_{j}^{k}|\cdot \text{exp}(j(\phi_{i}^{k}-\phi_{j}^{k}))\cdot \langle \left|U_{k}||U_{k}\right|\rangle \\ =\sum_{k}|K_{i}^{k}||K_{j}^{k}|\cdot \text{exp}(j(\phi_{i}^{k}-\phi_{j}^{k}))\cdot \gamma_{k}\\ =\sum_{k}g_{\mathrm{ij}}^{k}(\omega ,\theta )\\ g_{\mathrm{ij}}^{k}(\omega ,\theta )=|K_{i}^{k}||K_{j}^{k}|\cdot \text{exp}(j(\phi_{i}^{k}-\phi_{j}^{k}))\cdot \gamma_{k}=K_{i}^{k}\cdot K_{j}^{k*}\cdot \gamma_{k}\\ K_{i}^{k}(\omega ,\theta )=\int \kappa_{i}^{k}(t,\theta )e^{-j\omega t}dt \end{array}$$

Here, *ϕ*_*i*_^*k*^ = arg{*K*_*i*_^*k*^} is the phase-delay induced by the kernel mapping the *k*-th innovation to the *i*-th channel. Eq. (3) just means that the predicted cross-spectrum is a linear mixture of cross-spectra induced by each innovation. This mixture depends on the mapping from each innovation or source to the channels in question. For example, in local field potential recordings, the number of innovations and channels could be the same. However, in non-invasive electromagnetic studies, the number of channels can be much greater than the number of sources.

Eqs. (2) and (3) provide a generative model of sample cross-spectra. We have exploited this sort of model for steady-state responses extensively, when trying to infer the neuronal architectures generating local field potentials and other electromagnetic signals (Moran et al., 2007, 2008). However, these models used real-valued cross-spectra $\left|g_{\mathrm{ij}}(\omega )\right|\in R$, which ignore systematic phase-differences. So how do phase-differences induced by the transfer functions appear in complex cross-spectra? Eq. (3) shows that the cross-spectrum is a mixture of complex components due to each innovation, where the phase-differences *δ*_*ij*_^*k*^ = *ϕ*_*i*_^*k*^ − *ϕ*_*j*_^*k*^ caused by each innovation are weighted by the amplitudes |*K*_*i*_^*k*^| · |*K*_*j*_^*k*^| of the associated transfer functions. This means the phase of the cross-spectrum is a complicated mixture of phase-differences that is related to the average phase-difference between channels. The average phase-difference, induced by all the innovations together is:

4 δij=∫δijp(δij)dδij=∫δij(U)p(U)dUδij(U)=arg{Si}−arg{Sj}:mod2πSi(U)=∑k|Kik|·|Uk|exp(j(arg{Uk}+ϕik))p(U)=∏kp(Uk)pRe(Uk)=N(0,γk)pIm(Uk)=N(0,γk)

**Fig. 1:**
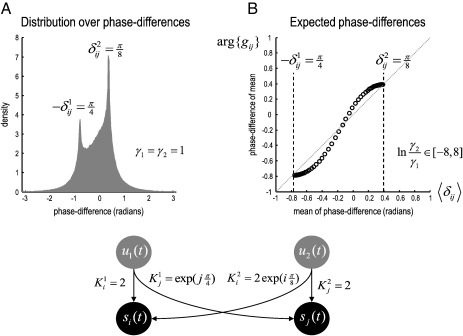
Phased distribution functions and expected phase-differences. Panel A shows the distribution over phase-differences between two channels or sources. In this (toy) example, we have introduced an asymmetry in the amplitude of the innovations driving each source (and the coupling between them). This results in a rather complicated distribution with two peaks corresponding to the phase-delays induced by the innovations at each source respectively. Panel B shows the relationship between the phase-difference of the (complex) cross-spectrum and the mean of the phase-difference. This relationship (dots) was disclosed by varying the relative amplitude of the innovations driving the sources. The lower panel details the simple form of the transfer functions assumed for this illustrative example.

This is a rather complicated integral to evaluate (involving Gauss hypergeometric forms; see Lee et al., 1994). Fig. 1 (left panel) shows the implicit density over phase-differences for a simple example with asymmetries in how innovations drive the signals. It can be seen that the density peaks at the phase-difference induced by each innovation. If we now plot the phase-delay function arg{*g*_*ij*_(*ω*, *θ*)} against the average phase-difference (the numerical integral in Eq. (4)), we see that the two are closely related (but are not the same because the phase of an average is not the average of a phase). Fig. 1 (right panel) shows the relationship (dots) between the phase of the cross-spectrum and the average phase-difference; this relationship was disclosed by varying the relative power of the two innovations, where ln *γ*_2_/*γ*_1_ ∈ [− 8, 8], while keeping the time-delays fixed.

Fig. 1 illustrates the key problem with interpreting phase-delays in terms of time-delays, when the sources of data are reciprocally coupled: the phase-delay function arg{*g*_*ij*_(*ω*, *θ*)} is zero for certain combinations of power. However, the time-delays did not change. Put simply, the phase-delays induced by different innovations can cancel each other out. Only when the power of one innovation predominates is the symmetry broken. In this situation, the phase-delay function reflects the time-delays associated with the larger innovation. This means that the phase-delay function is a lower bound on the phase-delays caused by each innovation (that depend on the time-delays between sources). This can be seen in the right panel of Fig. 1, which shows that phase-delays are bounded by the phase-differences induced by the two innovations. This means that the strength and time-delay of connections among distributed sources of data cannot be recovered from cross-spectra (or phase-delay functions) in the absence of a generative model that specifies how sources are connected.

#### Summary

In summary both the real and imaginary parts of cross-spectra contain useful information about the underlying system. The modulus relates to the measure of coherence, while the argument (phase or angle) is a complicated function of phase-delays induced by exogenous fluctuations (innovations). However, neither provides a unique or complete description of how data are generated and may be better thought of as data features that have yet to be explained by a generative model. In what follows, we will therefore consider the key data feature the sample cross-spectra *g*_*ij*_(*ω*) and treat both its real and imaginary parts on an equal footing. We will use conditional predictions of |*g*_*ij*_(*ω*, *θ*)|, arg{*g*_*ij*_(*ω*, *θ*)} and *FT*^− 1^{*g*_*ij*_(*ω*, *θ*)} to report the coherence, phase-delay and cross-correlation functions for pairs of hidden neuronal states and observed signals. To generate these predictions we need the system's kernels, *κ*_*i*_^*k*^(*τ*, *θ*). These are specified in a straightforward way by the form of the model and its parameters, as described next.

### From models to kernels

The kernels obtain analytically from the Jacobian I=∂f/∂x describing the stability of flow $\dot{x}=f(x,u,\theta )$ of hidden neuronal states, *x*(*t*) and a mapping (forward model) *s*(*x*, *θ*) : *x* → *s* that couples hidden states to observed signals (channel data). For channel *i*, and innovation *k*, the kernel (which can be evaluated numerically) is

5 $$\begin{array}{c} \kappa_{i}^{k}(\tau ,\theta )=\frac{\partial s_{i}(t)}{\partial u_{k}(t-\tau )}\\ =\frac{\partial s_{i}(t)}{\partial g(t)}\frac{\partial g(t)}{\partial x(t)}\frac{\partial x(t)}{\partial x(t-\tau )}\frac{\partial x(t-\tau )}{\partial \dot{x}(t-\tau )}\frac{\partial \dot{x}(t-\tau )}{\partial u_{k}(t-\tau )}\\ =\frac{\partial g_{i}}{\partial x}\text{exp}(I\tau )I^{-1}\frac{\partial f}{\partial u_{k}} \end{array}$$

**Fig. 2:**
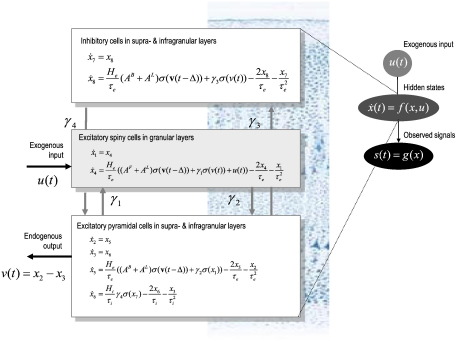
Equations of motion for a single source. This schematic summarizes the equations of motion or state equations that specify a neural mass model of a single source. This model contains three sub-populations, each loosely associated with a specific cortical layer; corresponding roughly to spiny stellate input cells, deep pyramidal output cells and inhibitory interneurons. Following Felleman and Van Essen (1991), we distinguish between forward connections (targeting spiny stellate cells in the granular layer), backward connections (with slower kinetics and targeting pyramidal cells and inhibitory interneurons in both supra- and infragranular layers) and lateral connections (targeting all subpopulations). The output of each source is modeled as a parameterized mixture of the depolarization of each subpopulation (primarily the pyramidal cells). The second-order differential equations describe changes in (vectors of) hidden states x(t) (e.g., voltage and current) that subtend observed local field potentials or EEG signals. These delay differential equations effectively mediate a linear convolution of presynaptic activity to produce postsynaptic depolarization v(t). Average firing rates within each sub-population are then transformed through a nonlinear (sigmoid) voltage-firing rate function *σ*(·) to provide inputs to other populations. These inputs are weighted by connection strengths. Here, **v**(*t* − Δ) represents a vector of (primarily) pyramidal depolarization in all sources, delayed by a connection-specific time-lag. Intrinsic connection strengths *γ*_*i*_: *i* = 1, …, 4 are shown connecting the different populations in different layers. When these equations are linearized around the system's fixed point, they specify the systems transfer function, and implicitly, the complex cross-spectra mapping from exogenous neuronal fluctuation or innovations u(t) to observed responses s(t). These functions depend on the parameters of the model that include the extrinsic connection strengths and other parameters like *H*_*j*_, *τ*_*j*_: *j* ∈ {*i*, *e*} that control post-synaptic responses of inhibitory and excitatory populations. Under assumptions about the spectral form of the innovations, this constitutes a generative model of observed cross-spectra over multiple channels.

This means the kernels are functions of the model's equations of motion and output mapping. The output mapping may be a simple gain function (for LFP data) or an electromagnetic forward model (for EEG and MEG data). The use of the chain rule follows from the fact that the only way past inputs can affect current outputs is through hidden states. The particular equations of motion used here correspond to a neural-mass model that has been used extensively in the causal modeling of electromagnetic data (David and Friston, 2003; Jansen and Rit, 1995; Moran et al., 2008). These equations implement a simple but biologically motivated (alpha-function based) model (Jansen and Rit, 1995) that captures the key aspects of synaptic processing; fast excitation and inhibition in layered cortical sources (Moran et al., 2011). The equations for a single source are summarized in Fig. 2.

#### Endogenous inputs

In a DCM comprising *N* sources, firing rates provide endogenous inputs from subpopulations that are intrinsic or extrinsic to the source (see Fig. 2). These firing rates are a sigmoid function of depolarization, which we approximate with a linear gain function (evaluated at the system's fixed point; Moran et al., 2007). Subpopulations within each source are coupled by intrinsic connections (with a conduction delay of 4 ms: Lumer et al. (1997)), whose strengths are parameterized by *γ* = {*γ*_1_, …, *γ*_5_} ⊂ *θ*. These intrinsic connections can arise from any subpopulation. Conversely, in accordance with cortical anatomy, extrinsic connections arise only from the excitatory pyramidal cells of other sources. The strengths of these connections are parameterized by the forward, backward and lateral extrinsic connection matrices; $A^{F}\in R^{\mathrm{NxN}}$, $A^{B}\in R^{\mathrm{NxN}}$ and $A^{L}\in R^{\mathrm{NxN}}$ respectively, with associated conduction delays $\Delta \in R^{\mathrm{NxN}}$.

#### Exogenous fluctuations

The innovations correspond to exogenous fluctuations $u(t)\in R^{Nx1}$ that excite the spiny stellate subpopulation in the granular layer. We parameterize their spectral density, *γ*(*ω*), in terms of white and pink spectral components; where these power law terms are ubiquitous features of neuronal noise (Freeman et al., 2003; Stevens, 1972):

6 $$\gamma_{k}(\omega )=\alpha_{u}+\frac{\beta_{u}}{\omega }$$

As noted above, one innovation is associated with each neuronal node or source.

#### Neuronal responses

The observer function is a mapping from *N* sources to observed data features expressed at *M* channels: $s(x,\theta )=L(\eta )\tilde{x}$, where $\tilde{x}(t)\in R^{Nx1}$ is a mixture of the depolarizations over subpopulations in each source. For invasive LFP recordings (that are obtained close to neuronal sources) this mapping can be reduced to a simple gain matrix, *L* = *diag*(exp(*η*_1_, …, *η*_*N*_)) where the parameters model electrode-specific log-gains. In EEG and MEG (electro- and magnetoencephalography) the mapping is specified with a gain matrix of lead-fields, $L(\eta )\in R^{\mathrm{MxN}}$, with unknown spatial parameters, *η* ⊂ *θ*, such as source location and orientation. Generally, this matrix rests upon the solution of a conventional electromagnetic forward model.

This completes the description of the neuronal model and, implicitly, the generative model for modeled cross-spectra. This model contains unknown parameters *θ* ⊃ {*γ*, *A*, *Δ*, *α*, *β*, *η*, …} controlling the strength and delays of intrinsic and extrinsic connections, the auto-spectra of innovations and the electromagnetic forward model. These parameters define the kernels and associated cross-spectra in Eq. (3). To complete our specification of a generative model, we presume the data (sample cross-spectra) to be a mixture of the predicted cross-spectra, channel noise and Gaussian prediction error (see Moran et al., 2009 for details)

7 gij(ω)=gij(ω,θ)+αs+βsω+εij(ω)Re(εij)~N(0,Π(ω)ε−1)Im(εij)~N(0,Π(ω)ε−1)

The channel noise, like the innovations, is parameterized in terms of (unknown) white (*α*) and pink (*β*) components, which can include channel-specific and non-specific components. Please see Moran et al. (2009) for more details.

#### Summary

This section has motivated the use of complex cross-spectra as data features that summarize the behavior of ergodic time-series. We have seen that only the absolute values of cross-spectra are used to form measures of coherence. Although coherence depends upon the dispersion of phase-differences, it is not sensitive to the expected or systematic phase-differences that could be introduced by neuronal dynamics or conduction delays. A simple solution to this is to use a generative model of both the real and imaginary parts of the cross-spectra and fit these predictions to sample cross-spectra. To do this we need a Bayesian model inversion scheme that can handle complex-valued data.

## Inverting models of complex-valued data

In this section, we consider a generalization of the variational scheme (Friston et al., 2007) used to invert Dynamic Causal Models that can handle complex-valued data. In what follows, we will briefly summarize the overall principles of model inversion and list the special differences that attend the analysis of complex-valued data.

Almost universally, the fitting or inversion of Dynamic Causal Models uses a variational free-energy bound on the log-evidence for a model *m* (see Friston et al., 2007 for details). This bound is optimized with respect to a variational density *q*(*θ*) (which we assume to be Gaussian) on unknown model parameters. By construction the free-energy bound ensures that when the variational density maximizes free-energy, it approximates the true posterior density over parameters: *q*(*θ*) ≈ *p*(*θ*|*y*, *m*). At the same time, the free-energy itself F(y,q)≈lnp(y|m) becomes a bound approximation to the log-evidence of the data. The (approximate) conditional density and (approximate) log-evidence are used for inference on parameter and model spaces, respectively.

Usually, one first compares different models (e.g., with and without particular connections) using their log-evidence and then turns to inferences on parameters, under the model selected (for an overview of procedures for inference on model structure and parameters in DCM, see Stephan et al., 2010). Here, we focus on the use of the conditional density, given a single model, which we assume has a Gaussian form q(θ)=N(μ,Σ). This density is quantified by the maximum *a posteriori* (MAP) value of the parameters *μ* (corresponding to their conditional mean or expectation) and their conditional covariance *Σ* (inverse precision) that encodes uncertainty about the estimates and their conditional dependencies. Crucially, the conditional mean *μ* or MAP estimate of the parameters implicitly defines the conditional estimate of the system's transfer functions *κ*_*i*_^*k*^(*τ*, *μ*) and through these, the modeled cross-spectra *g*_*ij*_(*ω*, *μ*) and associated functions. In other words, having optimized the model and parameters with respect to free-energy, we can recover all the conventional spectral characterizations, such as coherence, phase-delay and cross-correlation functions. However, these are not descriptive characterizations, but are mechanistically interpretable (in the context of the model). To access these summaries, we need to express the free-energy of the variational density in terms of complex-valued data.

### The free-energy of complex-valued data

The free-energy is the average of the log-likelihood and log-prior of the model under the variational density and its entropy (see Friston et al., 2007; Kiebel et al., 2008a,b). For nonlinear models, under Gaussian assumptions about the variational density and observation noise, this has a very simple form:

8 F=ln(p(g(ω),θ)−lnq(θ)q=G(μ)+12ln|∂μμG|G=−12Re(ε)TΠεRe(ε)−12Im(ε)TΠεIm(ε)−12υTΠνυ+12lnΠε+12lnΠνε=g(ω,μ)−g(ω)ν=μ−υ

Here, *g*(*ω*, *μ*) represents any nonlinear prediction or mapping from model parameters to data features (cf, Eq. (6)) and *ε*(*μ*) ∈ *C* are the corresponding prediction errors (i.e., discrepancies between the sampled and predicted cross-spectra). Similarly, *v*(*μ*) ∈ *C* are prediction errors on the parameters, in relation to their prior density $p(\theta |m)=N(\upsilon ,\Pi_{\nu }^{-1})$. For complex-valued data, we have to separate the real and imaginary parts of the implicit sum of squared prediction error in Eq. (8). This is because the sum of an absolute value is not the absolute value of a sum. This means the sum, implicit in the linear algebra above, has to be performed separately for real and imaginary parts. In a similar vein, the partial derivatives of the Gibb's energy G(μ) with respect to the parameters are again separated into real and imaginary parts:

9 ∂μG=−Re(∂με)TΠεRe(ε)−Im(∂με)TΠεIm(ε)−Πνυ∂μμG=−Re(∂με)TΠεRe(∂με)−Im(∂με)TΠεIm(∂με)−Πν

The gradients in Eq. (9) are used in a Gauss-Newton scheme to optimize the parameters iteratively, until the free-energy has been maximized. In practice, things are a little more complicated because one often makes a mean-field assumption when estimating parameters of the model and the noise precision, Π_*ε*_ (inverse covariance). In other words, the precision of the prediction error is usually assumed to be conditionally independent of the parameters. The gradient ascent then becomes a coordinate ascent that optimizes the conditional expectations of the model and precision parameters respectively. This is called Variational Laplace, which reduces to classical expectation maximization under some simplifying assumptions. A full description of these schemes, and their relationship to each other, can be found in Friston et al. (2007).

#### Summary

In this section, we have considered the central role of the free-energy bound on log-evidence used in model selection and inversion. The only thing we have to worry about, when dealing with complex-valued data, is to separate the real and imaginary parts of the data (and implicitly prediction errors), when evaluating the free-energy and its gradients. Having done this, we can then use standard schemes to optimize the parameters of any Dynamic Causal Model and select among competing models to find the one that has the highest free-energy (log-evidence). We now illustrate the application of this scheme using simulated data.

## Simulations and validation

In this section, we use simulated data from four sources, with known directed connections and delays, to establish the face validity of the inversion scheme of the previous section. Our particular focus here is on the improvement in the precision of parameter estimates, when including the phase information in complex cross-spectra. To illustrate this we will look closely at the conditional density over conduction delays. We will then be in a position to compare these estimates with true values and how these conduction delays translate into phase-delays and time-lags at the level of simulated population dynamics.

### Simulations

**Fig. 3:**
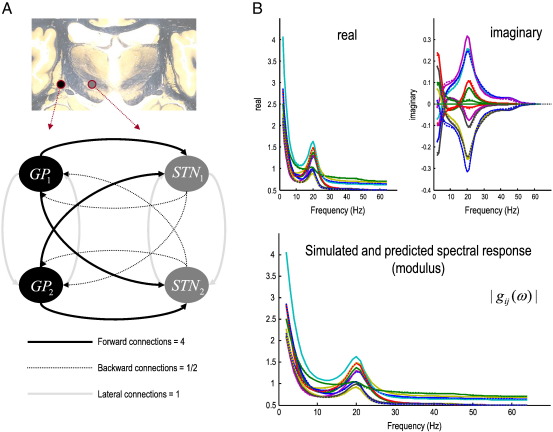
Estimated and predicted cross-spectra. Panel A shows the network or graph of sources used to simulate spectral data. Two pairs of sources are connected by forward, backward and lateral connections with strengths chosen to enforce directed (forward) connectivity. These simulated sources correspond to the sources of empirical LFP data (analyzed later) in the globus pallidus and subthalamic nucleus (shown figuratively on a coronal section of the human brain). B: Estimated and predicted cross-spectra for complex (upper panels) cross-spectral data and the corresponding absolute values or modulus of these data (lower panel). The auto and cross-spectra are shown for all four simulated channels as dotted (colored) lines. The predicted cross-spectra, following optimization of the model parameters, are shown as solid lines and illustrate the accuracy of model inversion.

To simulate data, we used the (David and Friston, 2003) neural mass model above to simulate four sources, organized into two pairs. The sources within each pair were coupled with lateral connections, whereas there was an asymmetric directed coupling between the first and second pair. This allowed us to look at predicted and estimated cross-spectra within and between pairs and illustrate the consequences of reciprocal connections between sources. The data were generated using the model parameters estimated from the empirical data of the next section. The only difference was that we suppressed backward connection to enforce an asymmetric (directed) coupling between the two pairs. The data were modeled as arising from pairs of sources in the Globus pallidus (GP) and subthalamic nucleus (STN). The connections from the GP to the STN constitute the forward (GABAergic) connections of the indirect basal ganglia pathway, while the reciprocal (glutamatergic) connections are from the STN to the GP. To suppress these backward connections we set them to a half, while the forward connections were given a value of four. The lateral connections were given intermediate values of one. This means our forward connections were 2 × 4 = 8 times stronger than the backward connections (i.e., the first pair of sources drove the second). The conduction delays were as estimated from the empirical data. See Fig. 3 (left panels) for a schematic of this small network of sources.

#### Simulating spectra

Data were simulated over 4 s with time bins of 4 ms. Cross-spectra were generated directly in frequency space, assuming that each source was driven by random fluctuations and that LFP data were observed with a signal to noise ratio of about 10%. The spectral characteristics of the innovations and channel noise were controlled by mixing white and pink noise components (see Eqs. (6) and (7)), using the conditional parameter estimates from the empirical analysis reported in the next section.

**Fig. 4:**
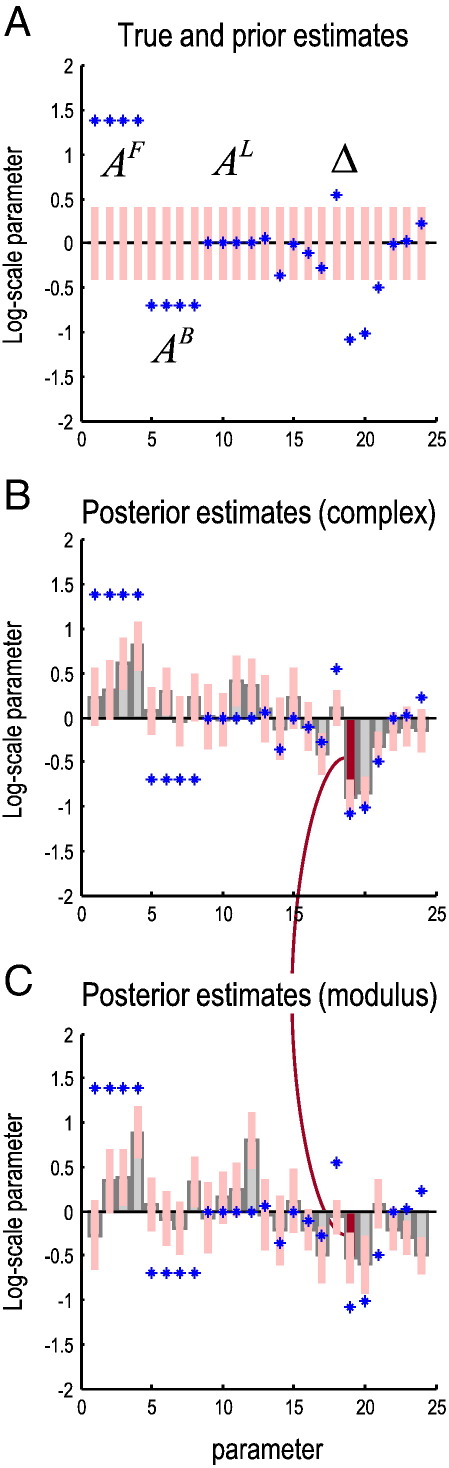
True and predicted parameters of the DCM. The upper panel (A) shows the true and prior values of key coupling parameters in the DCM of the previous figure, while the lower panels show the posterior or conditional densities after fitting the model to complex (B) and absolute (C) cross-spectra from four regions. Only the extrinsic (forward, backward and lateral) connection strengths (first twelve) and associated conduction delays (second twelve) are shown. These were the key model parameters that define the network architecture. The blue crosses are the true values and the pink bars correspond to 90% confidence intervals (prior confidence in A and posterior confidence in B and C). The conditional means are depicted as gray bars. These are the expected log-scale parameters that scale the connection strengths and delays. The true values disclose the asymmetry in this directed connectivity, which we hoped would reveal substantive phase-delays. *A priori*, the connection strengths and delays have log-scaling parameters of zero (i.e., are equal to their prior mean). The curved line highlights a conduction delay that is the focus of the next figure.

An example of these simulated data (sample cross-spectra) is shown in Fig. 3 (right panels) and illustrates the characteristic beta coupling seen in patients with Parkinson's disease and animal lesion models thereof (Lehmkuhle et al., 2009; Silberstein et al., 2005). These simulated cross-spectra were then used to invert the neural mass model described. Because connections strengths and time delays (and other model parameters) are nonnegative quantities, their prior mean is scaled by a free parameter with a log-normal distribution. We refer to these as the log-scale parameters, such that a log-scaling of zero returns the prior mean. Priors, $p(\theta |m)=N(\upsilon ,\Pi_{\nu }^{-1})$ (Eq. (8), above) are specified in terms of their prior mean *η* and variance *ζ* (as detailed in Moran et al., 2008 Table 1 and available in SPM8 http://www.fil.ion.ucl.ac.uk/spm). The prior variance determines how far the scale-parameter can move from its prior mean. Parameters, like the maximum excitatory potential and channel time constants have a prior variance of *ζ* = 1/8 : {*ζ* ∈ *Π*_*ν*_^− 1^}, allowing for a scaling up to a factor of about four. In contrast, relatively flat priors are used for effective connectivity measures (the parameters of interest) to allow for an order of magnitude scaling (with a prior variance of *ζ* = 1/2). This ensures that their posterior estimates are determined primarily by the data. In other words, the scheme will optimize the strength of all connections in the model, both intrinsic to each source (Fig. 2) and extrinsic between sources (Fig. 3). There is no bias in the estimates; however, the prior variances of the extrinsic parameters are larger than those of the intrinsic parameters, allowing for greater divergence from their prior mean in posterior estimation (c.f. Eq. (8)). We have chosen to highlight these extrinsic connectivity estimates in Fig. 4 because these quantify inter-regional coupling and determine the delays, coherence and phase at the source and sensor levels.

### Parameter estimates and their cross-spectra

Fig. 3 (right panels) shows the simulated sample cross spectra, in terms of their real and imaginary parts (upper panels) and the corresponding absolute values or modulus (lower panel). The auto and cross-spectra for all four simulated channels are shown as dotted (colored) lines. Following optimization of the model parameters, the modeled cross-spectra are shown as solid lines and illustrate the goodness of fit or accuracy of model inversion (they are barely distinguishable in many cases). The key thing to take from this example is the pronounced cross-spectral density in the beta range (20 Hz) that can be seen in both the real and imaginary parts. The relative contribution of the complex part is only about 10% of the real part but is concentrated in the frequency ranges over which coherence induced by coupling among the sources is expressed. The imaginary part of the complex cross-spectra (upper right panel) contains information that enables the estimation of phase-delays. These predicted cross-spectra are based on the conditional means of the parameters shown in the next figure.

The upper panel of Fig. 4 shows the true and prior values of the key coupling parameters in this DCM (for clarity, only the strengths and conduction delays of the extrinsic connections among sources are shown**)**. The lower two panels show the posterior or conditional densities after fitting the model to complex (middle panel) and absolute (lower panel) cross-spectra from the four regions. These estimates allow us to quantify any improvement in the accuracy or precision of parameter estimates, in relation to the true values, when inverting complex data relative to absolute data (Moran et al., 2009). The model comprises four forward connections (A^F^) from the GP to STN (Fig. 3), four backward connections (A^B^) from the STN to GP, and four lateral connections (A^L^). Given the conditional densities over these parameters, we can not only assess whether the complex scheme provides estimates that are closer to the true parameter values than the corresponding modulus-based estimates, but also whether the conditional precision or confidence increases. In the upper panel, we see that *a priori* all log-scale parameters are zero; these priors regularize the estimates and induce a “shrinkage” effect on the posterior estimates. The pink bars correspond to the prior 90% confidence interval. The true values of the simulated parameters are shown as blue crosses. The true strengths disclose the asymmetry in this directed connectivity, which we hoped would subtend substantial phase-delays.

#### Conditional densities over extrinsic connections

First we consider the first twelve parameters corresponding to the connections strengths, *A*^*F*^, *A*^*B*^ and *A*^*L*^. These are shown in the lower panels, where the gray bars report the posterior mean and pink bars denote 90% posterior confidence intervals. In the middle panel of Fig. 4, one can see that the asymmetry in the GP-STN network has been detected using the complex spectra, with larger values for the forward connections than for the backward connections. However, the shrinkage priors have precluded the forward connections from attaining their true values of log(4). Interestingly, the inversion has failed to decrease the backward connections to their true value of −log(2). These posterior densities should be compared with the lower panel in Fig. 4, illustrating the equivalent densities obtained after fitting the absolute cross-spectra. In this example, the conditional estimates using the complex and modulus schemes are roughly the same.

#### Conditional densities over conduction delays

However, Fig. 4 reveals a greater improvement in the estimation of the delays (Δ) for the complex compared to the modulus-data scheme. These are the second set of twelve parameter estimates. In particular, we observe that the 90% confidence intervals encompass the true simulated values in ten of the twelve parameters in the complex-scheme, compared to only seven of twelve parameters in the modulus scheme. Crucially, the estimates of the delays are more uncertain for conventional modulus-based schemes than when using complex-valued data. We now look at this more closely.

**Fig. 5:**
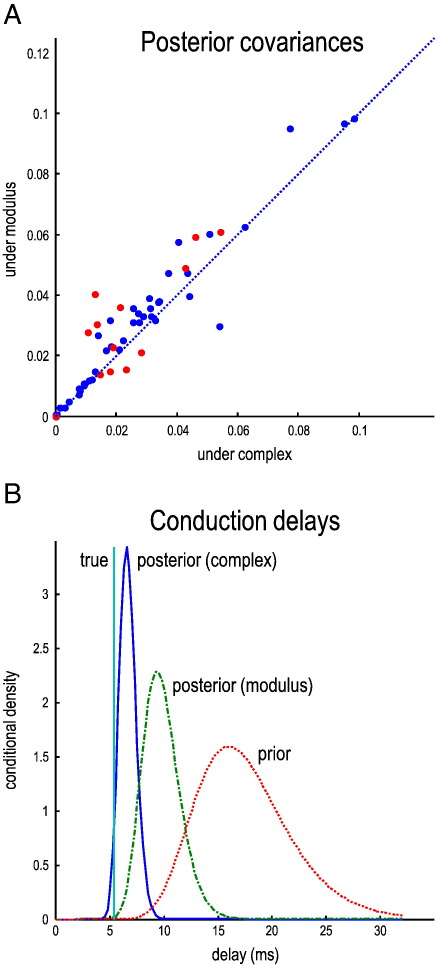
Conditional densities and conduction delays. Panel A shows the posterior uncertainty (covariance) for all (127) unknown or free parameters in this DCM. Here, we have plotted the conditional uncertainty (after fitting the absolute cross-spectra) against the equivalent uncertainty using complex cross-spectra. As might be anticipated, the uncertainties in the estimates are (in general) reduced when fitting complex-valued data features; i.e., most dots are above the identity line. These differences are particularly marked for the estimates of conduction delays, marked by the red dots. The bottom panel (B) provides the full conditional density on the conduction delays for one connection (the connection from the third source to the last). This is the parameter that showed the greatest change in conditional covariance under the two inversions. The true value of the condition delay was about 5 ms and falls within the posterior density, using complex-valued data (blue line). This is very distinct from the (broader) prior density shown in red. Interestingly, the posterior density obtained when using the absolute data has an intermediate value but fails to include the true value within its 90% confidence interval.

The upper panel of Fig. 5 shows the posterior uncertainty (covariance) for all (127) unknown or free parameters in this DCM. Here, we have plotted the conditional uncertainty after fitting the modulus data against the equivalent uncertainty when using complex cross-spectra. As might be anticipated, the uncertainties about estimates that are informed by the modulus only are higher than when both real and imaginary parts are used. These differences are particularly marked for the estimates of conduction delays (marked by red dots). In some instances, there has been more than a doubling of the conditional precision or certainty when using complex data. This is exactly the sort of behavior we hoped to observe and reflects the improvement afforded by generative models of complex data. The bottom panel provides the full conditional density on the conduction delay for one lateral (within pair) connection (the connection from the third source to the last). This is the parameter that showed the greatest change in conditional covariance under the two inversions (indicated by the connecting line in Fig. 4). The true value of the conduction delay was about 5 ms and falls within the posterior (shown in blue) when using complex-valued data. This is very distinct from the broader prior density shown in red. Interestingly, the posterior density obtained when using the modulus data has an intermediate value and fails to include the true value within its 90% posterior confidence interval. These results illustrate the increased accuracy and precision of posterior inferences, particularly on delay parameters, that are afforded by using complex-valued data with both real and imaginary parts.

We now turn to the implicit coherence, phase-delay and cross-correlation functions predicted by the parameter estimates. In what follows, we will actually use the cross-covariance functions to present quantitatively, the shared variance in two signals. Furthermore, we will divide the angular phase-delay by frequency and display it in units of milliseconds.

### Predicted coherence and phase-delay functions

**Fig. 6:**
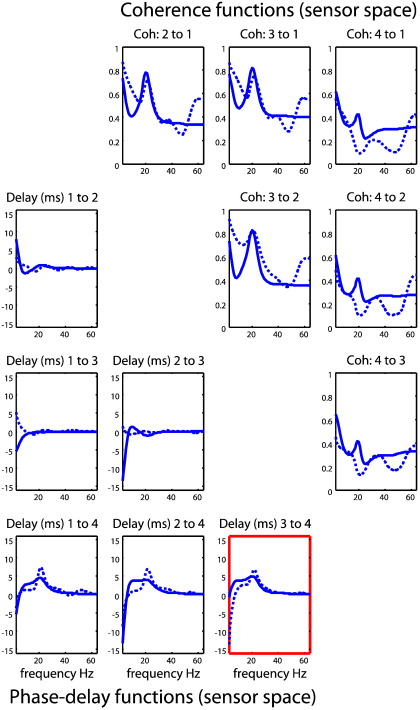
Observed and predicted coherence and phase-delays. The upper right panels show the observed (dotted lines) and predicted (solid lines) coherence among the four simulated sources. The panels on the lower left show the corresponding phase-delay functions in milliseconds. The coherence between one channel and a second is the same as the coherence between the second and the first. Conversely, the reciprocal phase-delay functions have the opposite sign.

The upper right panels in Fig. 6 show the sample (dotted lines) and modeled (solid lines) coherence among the four simulated channels. The panels below the leading diagonal show the corresponding phase-delay functions in milliseconds. The leading diagonal panels (pertaining to auto-spectra) have been omitted, because the associated phase-delay is zero and the coherence is one for all frequencies. Note that the coherence between one channel and another is the same (calculated as the angular phase-delay divided by frequency). There are two important points to be made using these results. First, there is a relatively poor correspondence between the sample and modeled coherence. This is because coherence (defined in Eq. (1); technically the magnitude squared coherence) is a highly nonlinear function of the original data features (the complex cross-spectra). From Eq. (1) we can see that the nonlinearity results from normalizing the absolute squared cross-spectra by the product of the auto-spectra from the two channels (Carter, 1987). It is possible that the large gamma coherence (~ 0.4 in some cases) observed from the channel data and not capitulated in our estimate result from unstable ratios at frequencies with low power in the auto-spectra. This contrasts with the modeled coherence based upon the modeled cross-spectra, which was produced by a biologically-motivated model (the DCM). The second point to note here is that the phase-delay functions are not constant over frequencies, despite the fact that the conduction delays were fixed during data generation. Like the coherences, the most interesting excursions are contained within the (beta) frequencies mediated by simulated interactions among the underlying sources. However, these are not estimates of conduction delays; as shown in the previous sections, they are lower bounds. For example, in the highlighted panel in Fig. 6 we see how the phase-delay function would suggest that this lateral (within-source pair) connection has a conduction delay of 5 ms, even though the reciprocal connections have different time-delays (5 ms for the connection from source 3 to 4, and 16 ms for the connection from source 4 to 3). This illustrates that there is no one-to-one mapping between the phase-delay and the underlying conduction delay. One can see this immediately by noting that in general the phase-delay (at any frequency) between two nodes is by definition anti-symmetric (a sign-reversal), even though there may be a greater conduction delay from one source to a second, compared with the conduction delay from the second to the first. The bottom line here is that it is extremely difficult to infer the direction of coupling or delays from phase-delay functions in the setting of reciprocal connections. In contrast, the conditional estimates of the parameters of the DCM afford an unambiguous characterization of conduction delays. We will pursue this in the next section but in the context of coupling among hidden sources, as opposed to channels.

### Frequency specific indices of coupling among channels and sources

**Fig. 7:**
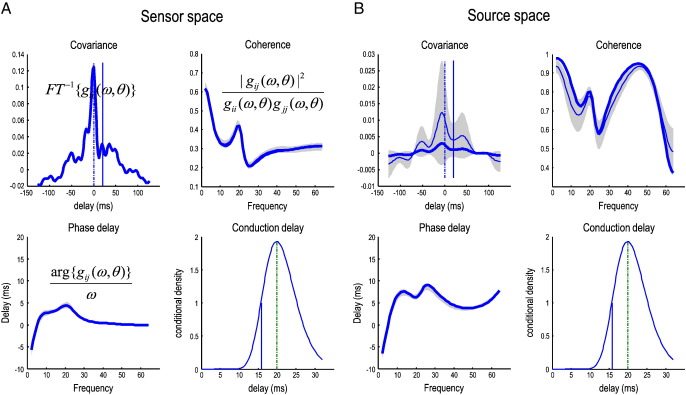
Indices of coupling among channels and sources. The panels on the left (A) describe the coupling between the first and fourth channels whereas the corresponding panels on the right (B) describe the same coupling between the first and fourth sources. For this pair of channels (resp. sources) the first panel shows the sample and modeled covariance as a function of lag in milliseconds. The second panel shows the corresponding coherence as a function of frequency and the third panel shows the phase-delay in milliseconds. Finally, the fourth panel shows the conditional density over the conduction delay associated with this connection. The solid blue lines represent the true (sample) values, the thinner blue lines correspond to the modeled values while the gray regions correspond to 90% confidence intervals and the vertical in the fourth panel is the true conduction delay. The covariance, coherence and phase-delay functions are all functions of the modeled cross-spectra, which depend upon the conditional means of the parameters of the generative model shown in previous figures. The functions in channel or sensor space (A) include both specific and nonspecific channel noise that has both white and colored components. In contrast, the source space functions are what would have been seen in the absence of noise (and unit gain on virtual LFP electrodes). This means the characterizations in source space are a mixture of neuronal and non-neuronal spectral features, whereas those on the right reflect the components or coupling due only to neuronal fluctuations or innovations.

To highlight the distinction between measures of coupling in channel and source space we will focus on a forward connection (between the first and fourth regions). Fig. 7 reports on this coupling between channels (left panels) and sources (right panels). For this pair of channels (resp. sources) the first panel shows the sample and modeled covariance as a function of lag in milliseconds (noting that the covariance function can be recapitulated in terms of a cross-correlation measure). The second panel shows the corresponding sample and modeled coherence as a function of frequency and the third panel shows the sample and modeled phase-delay in milliseconds as a function of frequency. Finally, the fourth panel shows the conditional density over its conduction delay. In all panels, the solid blue lines represent the true values used to generate the simulated data. The thin blue lines correspond to the conditional expectation, while the gray regions correspond to 90% posterior confidence intervals. The modeled covariance, coherence and phase-delay functions are all functions of the modeled cross-spectra, which depend upon the parameters of the generative model. In other words, there is a direct mapping from any set of parameter values to a particular covariance, coherence or phase-delay function. This means that we can compute the posterior confidence intervals simply by sampling parameters from the posterior or conditional density to produce a density on these functions. We have shown the conditional densities in channel and source space side by side to emphasize some key points.

The channel space characterizations include both specific and nonspecific instrumentation or channel noise that has both white and colored components (see Eq. (7)). In contrast, the source-space functions are what would have been observed in the absence of channel noise (and with unit gain on the LFP electrodes). This means the characterizations in channel space (left panel) are a mixture of neuronal and non-neuronal spectral features, whereas the source space results in the right panel reflect the components or coupling due only to neuronal fluctuations or innovations. Specifically, one can see that the modeled cross-covariance function in channel space is higher, tighter and estimated with a greater conditional confidence than the corresponding modeled and sample covariance function in source space. This is because the channel data contain a substantial amount of white noise that is common to all electrodes, resulting in a more peaked cross covariance function. When removed, one can see more clearly the underlying cross-covariances due to the neuronal fluctuations. These have a clear oscillatory pattern in the beta range (note the peaks around delays 50 and − 50 ms) that has been shaped by the neuronal transfer functions associated with each source. Similarly, the modeled and sample coherence in channel space are much smaller than in source space. This is due to the channel-specific noise component, which disperses the phase-differences and suppresses coherence. When this effect is removed, the coherence increases markedly, particularly at higher frequencies. In terms of phase and conduction-delays it can be seen that the modeled phase-delay increases, when considering sources in relation to channels. This effect can be explained in terms of nonspecific channel noise that changes the distribution of phase-differences, so that most of its probability mass is centered at zero lag. This means the (average) phase-delay shrinks towards zero (Daffertshofer and Stam, 2007). The implication here is that the phase-delay between channels represents a lower bound on the neuronal phase-delay between sources. For example, in the range 20–30 Hz, the phase-delay between channels does not exceed 5 ms, whereas it is nearly 10 ms between sources. Crucially, this is not the conduction delay (which would be the same for all frequencies). The true conduction delay in this example was ~ 15 ms and was estimated to be about 20 ms. Happily, the true value fell within the 90% conditional confidence interval (note that the conduction delays are the same for sensors and sources because they are an attribute of the underlying system not its measurement). It is also important to note that one could not deduce the conduction delay from peaks in the modeled or sample cross-covariance functions (zero and the conduction delays are shown as vertical lines). Although there is a small peak in the (sample and modeled) cross-covariance function between the two channels, there is no hint of such a peak in the modeled cross-covariance between sources. This speaks to the complicated relationship between the true (conduction) delay and how it is expressed both in terms of phase-delay functions and cross-covariance (and cross-correlation) functions (for an example from cortico-muscular recordings see Williams and Baker, 2009).

### Summary

In summary, we have used simulations to show that it is possible to recover the biophysical parameters of a reasonably realistic model of distributed responses from complex-valued data, summarized in terms of their sample cross-spectra. We have also seen that some parameters (especially conduction delays) are estimated more precisely when one uses complex cross-spectra, as opposed to its modulus. By identifying the system in terms of its parameters, one can derive coherence, phase-delay and other functions used in conventional measures of functional connectivity. However, it can be difficult to map back from these spectral characterizations to the architectures that caused them. In the next section, we consider an analysis of real data.

## Analyses of real data

**Fig. 8:**
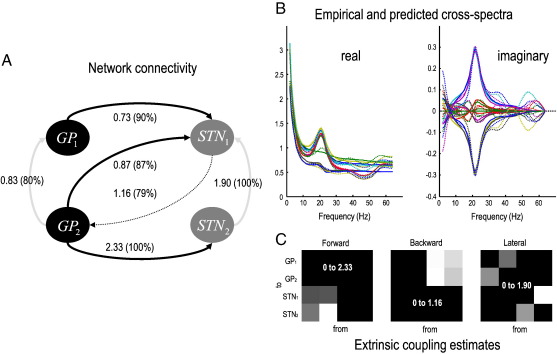
Parameter and state estimates using empirical data. A: Schematic showing the conditional estimates of coupling strengths among the four sources analyzed. We have only shown connections with a posterior probability (in brackets) of exceeding their prior mean was about 80%. This panel uses the same format as Fig. 3. B: These panels shows the real (left) and imaginary (right) predicted and observed data features (complex cross-spectra) using the real data from the four LFP channels described in the main text. As with the examples in Fig. 3 (using simulated data) the accuracy of these predictions is extremely high and captures most of the salient features in both the real and imaginary parts of these cross-spectra (with the exception of high frequencies). C: These panels shows the conditional estimates of the extrinsic coupling strengths for forward, backwards and lateral connections respectively (in the left, middle and right panels: The numbers over each panel specify the range of the grayscale used). The strongest connection was from the second globus pallidus source to the second subthalamic nucleus source. In terms of backward connections, the most prominent was from the first subthalamic to the second globus pallidus source. The corresponding predictions of this architecture, in terms of absolute cross-spectra are shown in the next figure.

**Fig. 9:**
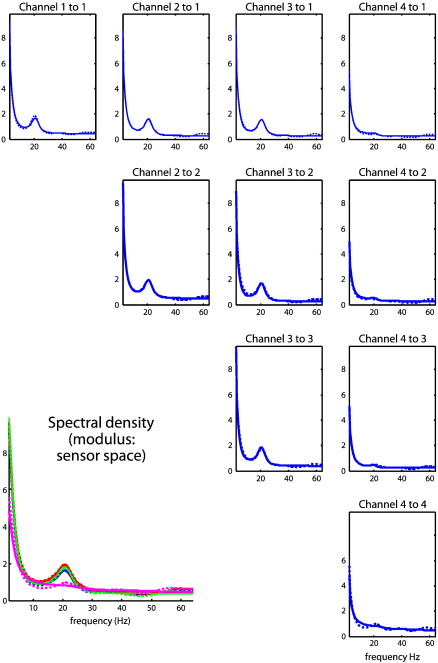
Predicted and observed cross-spectra for the empirical data. This figure shows the predicted (solid lines) and observed (dotted lines) absolute cross-spectra among the four empirical channels analyzed in the illustrative analyses. The auto-spectra occupy the leading diagonal and are gathered together on the lower left. In these data, we see a pronounced spectral density at 20 Hz, in most channels; although relatively suppressed in the cross-spectra involving the fourth channel. Again, these results show the high degree of accuracy seen in Fig. 8. The corresponding coherence and phase-delay functions are shown in the next figure.

**Fig. 10:**
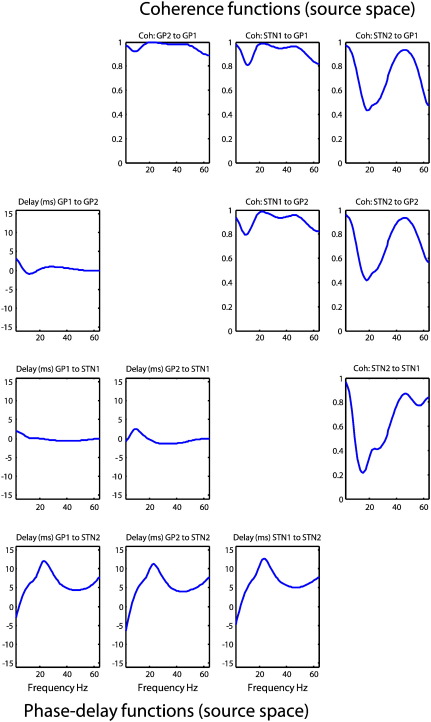
Coherence and phase-delay functions for empirical data. This figure uses the same format as Fig. 6 but presents the coherence and phase-delay functions following analysis of the real LFP data. Here, we have shown the coherences and phase-delays among sources, having removed channel noise. The most salient feature of these results is the marked phase-delay (more than 10 ms) between the second source in the STN and the remaining sources. Interestingly, the greatest coherence between this source and the remaining sources is seen in the high gamma range, whereas the beta coherence appears to be restricted to exchanges between the globus pallidus and the first subthalamic source.

**Fig. 11:**
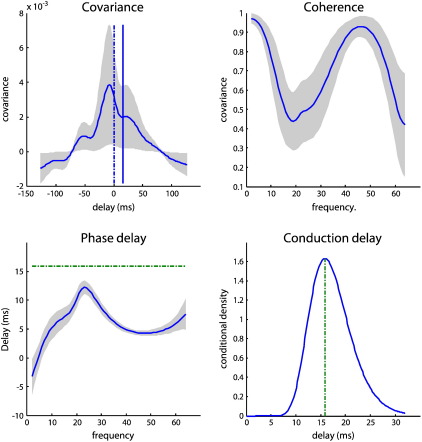
Coupling between the first and last sources. This figure uses the same display format as in Fig. 7 and shows the covariance, coherence and phase-delay functions for the connection between the first globus pallidus source and the second subthalamic nucleus source. Despite the reciprocal coupling between the sources, its asymmetry has induced a profound asymmetry in the covariance function, with greater covariances at lags up to about 30 ms. The resulting phase-delay function peaks at around 12 ms and is upper-bounded by the conditional estimate of the conduction delay (just above 15 ms). Notice that this is the value assumed for the simulated data in Fig. 7. Again, as with the examples using simulated data, there is a complicated and indirect relationship between the (estimated) conduction delay, phase-delay and peaks in the covariance function of lag.

In this section, we apply the analysis of the previous section to real data to demonstrate the reconstruction of conditional estimates of conventional measures of coupling among hidden sources and to highlight the complex relationship between these measures and underlying conduction delays. It should be noted that we are not presenting this analysis to draw any neurobiological conclusions but just to illustrate some technical points (an analysis of these data can be found in Mallet et al., 2008a,b). These data were acquired from adult male (6-OHDA-lesioned) Sprague–Dawley rats (Charles River, Margate, UK) in accordance with the Animals (Scientific Procedures) Act, 1986 (UK). Briefly, anesthesia was induced with 4% v/v isoflurane (Isoflo™, Schering-Plough Ltd., Welwyn Garden City, UK) in O_2_, and maintained with urethane (1.3 g/kg, i.p.; ethyl carbamate, Sigma, Poole, UK), and supplemental doses of ketamine (30 mg/kg, i.p.; Ketaset™, Willows Francis, Crawley, UK) and xylazine (3 mg/kg, i.p.; Rompun™, Bayer, Germany). Extracellular recordings of LFPs in the, external GP and STN were made simultaneously using ‘silicon probes’ (NeuroNexus Technologies, Ann Arbor, MI). Each probe had one or two vertical arrays of recording contacts (impedance of 0.9–1.3 MΩ measured at 1000 Hz; area of ~ 400 μm^2^). Neuronal activity was recorded during episodes of spontaneous ‘cortical activation’, defined according to ECoG activity. For the present paper, we used 4 s of data (downsampled to 250 Hz) from a single rat, comprising two (arbitrary) channels from the GP and STN probes. The cross-spectra were constructed from these time series using a vector autoregressive model (with order *p = 8* chosen to reflect the order of the neural state-space used in DCM see Moran et al., 2008). We then treated these empirical data in exactly the same way as the simulated data, i.e., we inverted the model with the same structure and priors as above. The results of this analysis are shown in Figs. 8 to 11, using the same format as for the simulated data.

### Spectral and parameter estimates

Fig. 8 shows the estimated extrinsic connections strengths and predicted data features (cross-spectra) using the real data from the four LFP channels described above. The free-energy objective function maximized during estimation (Eq. (8)) ensures maximum accuracy under complexity constraints, where complexity is the divergence between the prior and posterior densities (Penny et al., 2004). In other words, to avoid over-fitting, the model is constrained by priors over the parameters. In the present analyses, it is noteworthy that despite these constraints, the predictions in Fig. 8 are very accurate, capturing most of the salient features in both the real and imaginary parts of these cross-spectra (with the exception of frequencies above 50 Hz). The images (lower panels) show the conditional estimates of the extrinsic coupling strengths for forward, backward and lateral connections respectively (on the left, middle and right). The connection strengths and the posterior probability of exceeding their prior mean (of 32, 16 and 4 [arbitrary units] for forward, backward and lateral connections, respectively) are displayed alongside the connections in the left panel: The strongest connection was from the second pallidal source to the second subthalamic nucleus source. In terms of backward connections, the most prominent was from the first subthalamic to the second pallidal source (although both backward connections were weaker than their forward homologues). The most salient aspect of the ensuing architecture is a predominantly forward connectivity from GP to STN sources. This is consistent with the role of these connections in the indirect pathway. The predictions of this architecture, in terms of absolute cross-spectra, are shown in Fig. 9.

Fig. 9 shows the modeled (solid lines) and sample (dotted lines) absolute cross-spectra among the four channels. The auto-spectra along the leading diagonal are gathered together on the lower left. In these data, we see a pronounced spectral peak at 20 Hz in most channels; although relatively suppressed in the cross-spectra involving the fourth channel. The corresponding modeled coherence and phase-delay functions among the underlying sources are shown in Fig. 10. This figure follows the same format as Fig. 6 but presents the modeled coherence and phase-delay functions in source space (as opposed to sensor space) having removed channel noise. The most salient feature of these results is the marked phase-delay (more than 10 ms) between the second STN source and the remaining sources. Interestingly, the greatest coherence between this source and the remaining sources is seen in the gamma range (40–60 Hz in these data), whereas beta (20 Hz) coherence appears to be restricted to exchanges between the globus pallidus and first subthalamic source. In some cases these sources appear to have coherence approaching one. Fig. 11 uses the same display format as in Fig. 7 and shows the covariance, coherence and phase-delay functions for the connection between the first globus pallidus source and the second subthalamic nucleus source. The asymmetry in this bidirectional coupling has induced a profound asymmetry in the modeled cross-covariance function, with greater covariances at lags up to about 30 ms. The modeled phase-delay function peaks at around 12 ms and is upper-bounded by the conditional estimate of the (forward) conduction delay (just above 15 ms). From a linear systems perspective, the coupling here appears to be mediated by gamma coherence (upper right panel). This is consistent with the (asymmetrical) peaks of the cross-covariance function, where the first peak (for positive lag) occurs around 25 ms: this lag is not inconsistent with the high coherence at 40 Hz shown on the upper right. However, it would be a mistake to interpret these results as showing that signals from the GP to the STN source are delayed by 25 ms. Furthermore, the differential phase-delay (of 12 ms) in the beta range and (5 ms) in the gamma range does not suggest that fast frequencies are propagated with a smaller conduction delay than slow frequencies: The conduction delay is the same for all frequencies (about 15 ms). The frequency dependency of phase-delays is a result of interactions within and between sources, modeled here in terms of linear differential equations.

### Summary

In this section, we have applied DCM for complex cross-spectra to LFP recordings from subcortical sources that are constituents of the (indirect) cortico-basal ganglia-thalamic pathways. The conditional estimates of connection strengths suggest that the extrinsic (between-region) coupling was asymmetrical and directed, consistently for the two pairs of sources considered. The associated conduction delays were fairly long (15 ms), which may reflect real axonal propagation delays or the inherent slowness of population dynamics in relation to the firing of individual neurons. The modeled responses to neuronal fluctuations produced some interesting and complicated spectral behaviors; with the coherence between two sources being mediated largely by gamma coherence with a phase-delay that was substantially less than the phase-delay at beta frequencies predominant in the source region. This again highlights the complicated mapping between the underlying functional architecture generating signals and classical measures based on linear systems theory.

## Discussion

In summary, we have described a way to access conditional estimates of coherence, phase-delay and covariance functions between time-series in sensor or source space. This rests on the inversion of Dynamic Causal Models as generative models for complex-valued data. The benefits of a generative model include the ability to see how various model parameters effect coherence and phase-delays in a frequency-specific manner. Furthermore, one can reconstitute the conditional coherence and related functions, not just between data channels but between any hidden states that are included in the model. In the examples above, we were able to look at the phase-delays between specific sub-populations comprising one of several sources of local field potential data. An important consequence of this is that we can access conditional coherence and phase-delay functions among sources that are observed non-invasively with EEG and MEG.

One important conclusion of this work is that one should be careful in interpreting estimates of phase-delays as an expression of conduction delays. Conduction delays are undoubtedly of major importance for understanding large-scale neuronal dynamics (Breakspear et al., 2006; Deco et al., 2009; Jirsa and Ding, 2004; Roberts and Robinson, 2008), and it is tempting to infer them by assuming a direct relationship with phase differences in recorded signals (e.g., Brazier, 1972; Gotman, 1981). However, as shown above, the relation between phase differences and conduction delays is not straightforward: phase-delays can differ across frequencies, while conduction delays are determined by axonal micro-architecture and are fixed across all frequencies. Furthermore, as shown in Fig. 7, conduction delays cannot be inferred from peaks in the cross-covariance function. In summary, inference on conduction delays can only be made with a model that parameterizes these delays. As suggested by our analyses (Fig. 4), inference on conduction delays can benefit from modeling the imaginary components of recorded data.

In this paper, we used invasive LFP recordings, assuming that the signal at each channel was provided by one source. However, exactly the same scheme can be applied to EEG and MEG data, where there may be many more (or less) channels than sources. In this context, the ability to recover conditional estimates of coupling among sources (as opposed to channels) is crucial and finesses some of the issues associated with interpreting coherence among channels, e.g., volume conduction effects (Schoffelen and Gross, 2009; Stam et al., 2007; Winter et al., 2007) or correlated noise in the context of Granger causal estimates (see Valdes-Sosa et al., 2011 for a discussion).

### Phase synchronization and Granger causality

In principle, any metric that has proven fruitful for connectivity analyses at the sensor level (such as phase-synchronization, transfer entropy or Granger-causal measures; e.g., Brovelli et al., 2004; Bressler et al., 2007; Dhamala et al., 2008; Lachaux et al., 1999; Rodriguez et al., 1999; Vakorin et al., 2010; Varela et al., 2001) can be derived from the conditional estimates provided by DCM. This is because conventional measures can be derived from the transfer functions that are determined uniquely by the parameters of a biophysical DCM. With the developments described in this paper, it is now possible to reproduce conventional metrics of coupling by replacing the conventional model-free (sample) estimator with a model-based (conditional) estimator. Crucially, this can be done in either sensor or source space.

Phase-synchronization is usually used to quantify the amount of nonlinear coupling between channels (e.g., Rosenblum et al., 1996; Tass and Haken, 1996). The phase-synchronization index (Eq. (2)) can be computed from the distribution of phase-differences (Eq. (4)), which is specified by the conditional estimates of a DCM. However, the underlying DCM can be linearized (as in this paper), which provides an interesting perspective on phase-synchronization. Many people (including ourselves; Chawla et al., 2001) have tried to understand how zero-lag phase-synchronization can emerge in nonlinear coupled neuronal oscillators. However, the linear systems perspective provides a rather trivial explanation: the phase-delays induced by random fluctuations that are passed between reciprocally connected sources cancel. In fact, it is rather hard to generate non-zero lag phase synchronization unless one introduces substantial asymmetries in the coupling (see Fig. 1). Whether this is a useful perspective remains to be established, particularly in the context of DCMs that model nonlinear coupling (e.g., Chen et al., 2008).

It is hoped that these developments may harmonize DCM and conventional time-series analysis. This is meant in the sense that conventional analyses in electrophysiology can now be complemented with conditional estimates of spectral behavior that are informed by the neuronal architecture generating these behaviors. This should allow intuitions about how phase relationships and coherence arise to be tested. The marriage between conventional (linear systems) time-series analysis and DCM is evident in this work at two levels. First, we use a linearization around the fixed point of the system to enable the use of linear systems theory to generate predicted spectral responses. Secondly, the data features predicted are themselves motivated by linear systems theory. However, the estimation of these sample spectra highlights the fundamental difference between the spectral characterizations used in conventional analyses and those furnished by DCM. This difference rests upon the underlying generative model. Our sample spectral data were constructed from time series using a vector autoregressive model At this point conventional approaches would stop and report power, coherence, and other metrics of functional connectivity and interpret these quantities directly. However, from the point of view of DCM, this autoregressive process (or spectral estimates derived from Fourier or wavelet based techniques) serves as a feature selection step to provide a compact summary of the data in terms of their sample cross spectra. The desired spectral estimates are those that are conditioned upon a biologically plausible DCM, which best accounts for the sample (conventional) spectra. In short, the difference between conventional and conditional cross-spectra (in sensor space) is that the latter are constrained by a model that allows one to put formal constraints and prior beliefs into the estimation. Furthermore, there is a unique mapping between the parameters of the underlying model and the conditional spectra provided by DCM. Employing complex-valued data features, as we have shown, becomes especially important when trying to establish spectral asymmetries in reciprocal connections (e.g. between forward and backward message-passing in the brain) and associating these asymmetries with the laminar specificity of forward and backward connections. To address these sorts of issues it will be necessary to examine conditional coherence between different subpopulations (i.e., cortical layers), which is, in principle, possible with DCM. We will pursue this in future work using ECoG recordings in awake-behaving monkeys (Rubehn et al., 2009).

## Conclusion

Perhaps the simplest and most important point made by the analyses in this paper is that conventional characterizations of coupling among observed channel data are basically the starting point for Dynamic Causal Modeling. In other words, we are interested in establishing how particular data features like coherence and phase-delay are generated biophysically. Once one has an explicit mapping between the underlying biophysical parameters of a generative model and the predicted behavior in terms of cross-spectral density (and associated functions) the rather complicated relationship between conduction strengths and delays and how they manifest in terms of coherence and cross-correlation functions becomes more evident. In this sense, Dynamic Causal Modeling of observed cross-spectra may allow one to further qualify and understand the subtleties of conventional summaries. Perhaps one of the most important (and unforeseen) aspects of the analyses presented here was how channel noise can influence sample covariance and coherence functions in such a qualitative fashion. One of the key advantages of having a generative model is that one can partition observed coherence into those parts that are mediated neuronally and those parts which are not. This may represent one step towards a more quantitative assessment of coherence and phase-delays and how they relate to asymmetries in the strength and conduction delays of underlying neuronal connections.

## Software note

All the inversion schemes and DCM analyses described in this paper can be implemented using Matlab routines that are available as part of our academic freeware from http://www.fil.ion.ucl.ac.uk/spm/software/spm8/.
